# A High Protein Diet Is More Effective in Improving Insulin Resistance and Glycemic Variability Compared to a Mediterranean Diet—A Cross-Over Controlled Inpatient Dietary Study

**DOI:** 10.3390/nu13124380

**Published:** 2021-12-07

**Authors:** Francesca Tettamanzi, Vincenzo Bagnardi, Panayiotis Louca, Ana Nogal, Gianna Serafina Monti, Sara P. Mambrini, Elisa Lucchetti, Sabrina Maestrini, Silvia Mazza, Ana Rodriguez-Mateos, Massimo Scacchi, Ana M. Valdes, Cecilia Invitti, Cristina Menni

**Affiliations:** 1 Department of Biomedical Sciences, Humanitas University, 20072 Pieve Emanuele, Italy; francesca.tettamanzi@st.hunimed.eu; 2 Department of Statistics and Quantitative Methods, University of Milan-Bicocca, 20126 Milan, Italy; vincenzo.bagnardi@unimib.it; 3 Department of Twin Research, King’s College London, St Thomas’ Hospital Campus, London SE1 7EH, UK; panayiotis.louca@kcl.ac.uk (P.L.); ana.nogal_macho@kcl.ac.uk (A.N.); 4 Department of Economics Management and Statistics, University of Milan-Bicocca, 20126 Milan, Italy; gianna.monti@unimib.it; 5 Laboratory of Metabolic Research, Istituto Auxologico Italiano, IRCCS, S. Giuseppe Hospital, 28824 Piancavallo, Italy; s.mambrini@auxologico.it (S.P.M.); s.mazza@auxologico.it (S.M.); massimo.scacchi@unimi.it (M.S.); 6 International Center for the Assessment of Nutritional Status (ICANS), Department of Food, Environmental and Nutritional Sciences (DeFENS), University of Milan, 20100 Milan, Italy; 7 Istituto Auxologico Italiano, IRCCS, S. Giuseppe Hospital, 28824 Piancavallo, Italy; e.lucchetti@auxologico.it (E.L.); s.maestrini@auxologico.it (S.M.); 8 Department of Nutritional Sciences, King’s College London, Franklin Wilkins Building, London SE1 9NH, UK; ana.rodriguez-mateos@kcl.ac.uk; 9 Department of Clinical Sciences and Community Health, University of Milan, 20122 Milan, Italy; 10 Inflammation, Injury and Recovery Sciences, School of Medicine, University of Nottingham, Nottingham NG5 1PB, UK; ana.valdes@nottigham.ac.uk; 11 Laboratory of Research in Preventive Medicine, IRCCS Istituto Auxologico Italiano, 20100 Milan, Italy; invitti@auxologico.it

**Keywords:** high protein diet, Mediterranean diet, insulin resistance, glycemic variability, obesity, gut microbiome, dietary intervention

## Abstract

The optimal dietary pattern to improve metabolic function remains elusive. In a 21-day randomized controlled inpatient crossover feeding trial of 20 insulin-resistant obese women, we assessed the extent to which two isocaloric dietary interventions—Mediterranean (M) and high protein (HP)—improved metabolic parameters. Obese women were assigned to one of the following dietary sequences: M–HP or HP–M. Cardiometabolic parameters, body weight, glucose monitoring and gut microbiome composition were assessed. Sixteen women completed the study. Compared to the M diet, the HP diet was more effective in (i) reducing insulin resistance (insulin: Beta (95% CI) = −6.98 (−12.30, −1.65) µIU/mL, *p* = 0.01; HOMA-IR: −1.78 (95% CI: −3.03, −0.52), *p* = 9 × 10^−3^); and (ii) improving glycemic variability (−3.13 (−4.60, −1.67) mg/dL, *p* = 4 × 10^−4^), a risk factor for T2D development. We then identified a panel of 10 microbial genera predictive of the difference in glycemic variability between the two diets. These include the genera *Coprococcus* and *Lachnoclostridium*, previously associated with glucose homeostasis and insulin resistance. Our results suggest that morbidly obese women with insulin resistance can achieve better control of insulin resistance and glycemic variability on a high HP diet compared to an M diet.

## 1. Introduction

An obesity pandemic is gripping the globe, with higher demand and availability for energy-dense foods, accompanied by increasingly sedentary lifestyles [1,2,3]. This is a major public health concern, as obesity often confers an increased risk of developing a wide range of complex and life-changing diseases, including cardiovascular and cerebrovascular disease, type II diabetes and cancers [4,5,6,7]. Therefore, the development and implementation of effective and affordable measures to combat obesity is of utmost importance. As well as encouraging increased physical activity, many efforts to reduce obesity and its associated disorders have focused on the impact of diet and nutrition [8]. In particular, the Mediterranean (M) diet, a diet characterized by high levels of polyphenols, mono- and polyunsaturated fatty acids (MUFAs and PUFAs), antioxidants, and fiber, as well as low levels of salt, sugar and saturated fatty acids [9], has been associated with improved health outcomes [9]. Greater adherence to the M diet has been associated with reduced risk of cardiovascular disease [9,10], which also supports weight loss [11]. A high-protein (HP) diet, comprising low carbohydrate, high fat and high protein intake, has also been suggested as a potential dietary intervention for obesity prevention [12] with HP diets corresponding to greater weight loss compared to similar isocaloric diets with standard protein content [13]. A HP diet has also been shown to lead to a greater weight loss compared to a high-carbohydrate diet, along with an improvement in insulin parameters, highlighting its power to lower the risk of type 2 diabetes and cardiovascular diseases [14,15]. Over the short term, a HP diet has been suggested to more effectively aid weight loss in contrast to a low-fat diet, and has been shown to change body composition in overweight or obese men [14]. The mechanisms supporting the HP diets’ effects on weight loss efficacy is theorized to be related to increased satiety [16] and it has been suggested that this enhances an individual’s metabolic rate [17]. Recent evidence suggests that the benefits of any dietary intervention are intrinsically linked to an individual’s metabolic profile [18]. The exact role of the gut microbiome in nutrient metabolism is still unclear, but various studies have linked microbial diversity and specific bacteria to a propensity for obesity, as well as to the metabolism of dietary compounds found in the M diet, including omega-3 fatty acids and polyphenols [19,20]. Here, we aimed to explore the differential effects on metabolic parameters elicited by the M and HP diets. As these effects are reported to be exacerbated in obese individuals with impaired metabolic response, we conducted a 21-day randomized crossover controlled dietary trial in 20 insulin-resistant women with obesity.

## 2. Materials and Methods

**Figure 1:**
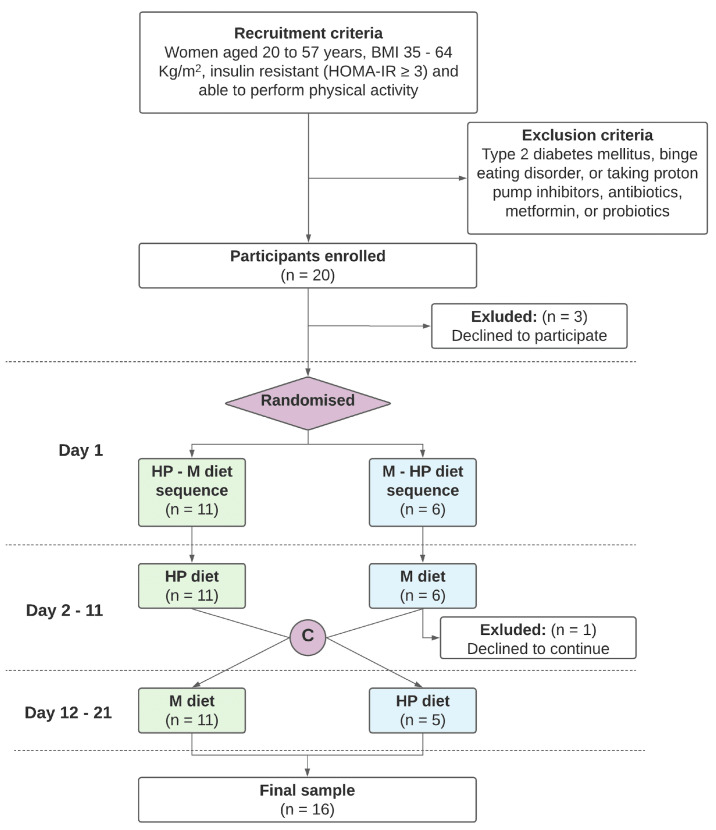
Study flowchart.

A flowchart of the study design is presented in Figure 1.

### 2.1. Study Design and Participants

This is an open-label, single-center randomized crossover controlled dietary trial in an inpatient setting. Participants were assigned to receive, in a 1:1 ratio, one of the two following dietary sequences: hypocaloric M diet followed by hypocaloric HP diet (sequence M–HP) or vice versa (sequence HP–M). Each period of intervention lasted 10 days with no washout before the switch from the first to the second diet. Participants were at San Giuseppe Hospital, Piancavallo of the IRCCS Istituto Auxologico Italiano, where they were hospitalized throughout the duration of the trial. Patients eligible for the study were women aged 20 to 57 years with BMI 35–64 kg/m^2^, insulin resistant (HOMA-IR ≥ 3) and able to perform physical activity. Exclusion criteria included individuals suffering from type 2 diabetes mellitus (defined by the presence of occasional plasma glucose value of ≥200 mg/dL or a fasting plasma glucose of ≥126 mg/dl or an HbA1c ≥ 6.5% (≥48 mmol/mol Hb)), binge eating disorder, taking proton pump inhibitors, antibiotics, metformin or probiotics. Moreover, women included in the study were not following any specific dietary patterns in the 6 months preceding study enrolment, and were characterized by prandial hyperphagia, excessive carbohydrates, lipid and sodium consumption, poor fiber intake and insufficient hydration.

The study protocol was approved by the Institutional Ethical Committee (2018_01_30_02) and all participants provided written informed consent before the trial.

### 2.2. Study Procedures

Eligible patients were randomly allocated into two groups on day 1: sequence M–HP or sequence HP–M. The M diet was composed of approximately 55% carbohydrates (whole wheat), 25% fat (PUFA from olive oil, almonds and pistachios) and 20% protein (fish, goat cheese and legumes). The HP diet was composed of approximately 40% carbohydrate, 30% fat and 30% protein. Both diets had the same caloric intake, which was 500 Kcal less than the individual daily caloric requirement, and a similar equally moderate glycemic load ranging between 11 and 19. Moreover, for both the M and HP diets, the energy derived from the consumption of simple carbohydrates (represented mainly by fruits and dairy products) was lower than 15% of the total energy. Animal and vegetable proteins were provided in both diets. In the M diet, protein consumption was in line with the relevant Food Guide Pyramid. Second courses included mainly white meat, bluefish, goat cheese and legumes. In the HP diet, proteins sources were mainly white meat, fish and eggs (Table S1).

On day 1, baseline measurements of clinical variables were obtained for each participant, including height, weight, waist and hip circumference, blood pressure, heart rate and body composition as measured by phase-sensitive, single-frequency bioimpedance analyzer (BIA 101, Akern, Pisa, Italy). Resting energy expenditure (REE) was assessed with indirect computerized calorimetry (Vmax 29, Sensor Medics, Yorba Linda, CA, USA), and the total energy expenditure (TEE) was estimated by multiplying the REE by Physical Activity Level (PAL), which was 1.2 for all, i.e., *TEE* = *REE* × 1.2. 500 kcal were subtracted from the individual TEE to determine the diet hypocaloric target. Additionally, fasting blood samples for insulin and lipids (total, LDL and HDL cholesterol, triglycerides) measurement and stool samples were collected. Fecal samples were immediately frozen at −20 °C. For the gut microbiota analysis, samples were stored at −80 °C directly until processing following 3–5 h refrigeration.

Measurements and sample collections were repeated during clinical visits on days 6, 11, 16, and 21. Adherence to the diet was closely monitored by the nurses. Throughout the study, glucose levels were monitored by flash continuous glucose monitoring (FSL-FGM; Free-Style Libre™; Abbott, Witney, Oxfordshire, UK).

### 2.3. 16S rRNA Gut Microbiome

Microbial 16S rRNA gene was extracted from fecal samples and sequenced using the Illumina MiSeq platform at the Genetic Laboratory, Erasmus Medical Centre in Rotterdam, the Netherlands. The Microbiota pipeline 25 was used to filter and cluster reads into Operational Taxonomic Units (OTU) based on 97% similarity against the SILVA database v132 [20,21]. Microbial diversity indices were calculated using the platform QIIME 2 (v2018.11) as the average value after rarefying the OTU table to 13678 reads. Shannon and Simpson indices were calculated to describe the alpha diversity (i.e., microbial diversity within individual samples) [22,23]:

$Shannon\;Index=-\sum_{i=1}^{s}\left(p_{i}log_{2}p_{i}\right)$, where s is the number of OTUs and *p_i_* the proportion of the community represented by OTU i.

$Simpson\;Index=1-\sum p_{i}^{2}$, where *p_i_* is the proportion of the community represented by OTU i.

### 2.4. Study Outcomes

The primary study outcomes were insulin and HOMA-IR measured as the change from baseline concentration during each diet (i.e., the difference between insulin, HOMA-IR from day 11 to day 1 for the first diet in the sequence, and between day 21 and day 11 for the second diet in the sequence) and glycemic variability. Individual HOMA-IR was computed as *HOMA-IR* = (*fasting insulin* × *fasting glucose*)/405 with glucose measured in mg/dl and insulin in µU/L.

Individual glycemic variability was measured for each diet as daily mean standard deviation (SD) of glucose concentration during continuous monitoring, that is, for each individual the mean standard deviation was calculated as $\underline{SD}=1/dx\;\sum SD_{d}$, where *SD_d_* is standard deviation of each day’s glucose measurements in HP or M diet. For overall evaluation of CGM data, we also computed (i) the difference between diets in mean blood glucose concentration and (ii) the percentage of time of sensor glucose concentration within target range (within 70–180 mg/dL), in hypoglycemic (below 70 mg/dL) and hyperglycemic (above 180 mg/dl) conditions. All CGM metrics were calculated using the R package iglu [24]. Secondary study outcomes included change from baseline in weight waist to hip ratio, fat to lean mass ratio, lipids, blood pressure, heart rate and microbial diversity metrics.

### 2.5. Statistical Analysis

The analysis dataset included all participants who completed the dietary sequence and had measurement of the main study outcomes at least at the beginning and at the end of each intervention period (i.e., day 1, 11 and 21) (Figure 1). According to the intention-to-treat principle, patients were analyzed in the dietary sequence assigned. Baseline characteristics of the study population were described as mean values along with their standard deviations (SD). To adjust for treatment period and sequence, a linear mixed effect regression model was fitted, which included as fixed predictors treatment type (HP over M), treatment period (P1 over 2) and sequence (HP–M over M–HP). The effect of the type of diet on glycemic variability was evaluated by calculating the difference in SD of glucose concentration between HP and M diets, and applying linear mixed effect regression model as described above. Additional study outcomes, including clinical, microbial and other glucose-related variables, were similarly analyzed. Estimates of unadjusted and adjusted mean values along with their 95% Confidence Intervals (CI) were calculated for each outcome. Mean differences and CI were standardized to obtain comparable effect sizes for considered variables, and were represented in forest plots.

Exploratory sub-analyses were performed to evaluate the association of baseline microbial taxa with the difference between HP and M diet in individual glucose variability (${\underline{SD}}_{HP}-{\underline{SD}}_{M}$, with SD as defined above), by using a Lasso regression model with zero sum constraint to account for the compositional nature of microbial data [25]. Relative abundances (RA) of OTU agglomerated to genus level were calculated, filtered if sparse in less than 80% of the samples, and log transformed before the analysis. For variable selection, 5-fold cross-validation was applied to tune the regularization term lambda. Associations were expressed as beta regression coefficients.

Statistical analyses were performed using the statistical software SAS 9.4 (SAS Institute, Cary, NC, USA) and R version 3.6.2. A *p*-value less than 0.05 was considered statistically significant.

## 3. Results

Between April and December 2018, 20 patients were enrolled in the study at the Piancavallo Hospital, Italy. Of them, three patients decided not to take part in the study before randomization, and one patient assigned to sequence M–HP discontinued the study after the first diet; 16 participants completed the dietary sequence assigned and represented the analysis set (Figure 1). As depicted in Figure 1, participants were enrolled in the study and randomly allocated on day 1 to one of two dietary sequences: the HP–M indicates high protein diet followed by Mediterranean diet, and the M–HP indicates the Mediterranean diet followed by high protein diet. Crossover (C) to the second diet occurred on day 12.

The characteristics of the participants at study entry are presented in Table 1. On average, women were slightly younger, had a lower BMI, fasting glucose, insulin and HOMA-IR in the HP–M group compared to the M–HP group. However, differences were not statistically significant.

The HP and M diets led to a similar loss of body weight, with a mean change from baseline of −2.71 (95% CI: −3.59, −1.82) kg and −2.09 (95% CI: −2.71, −1.46) kg, respectively (Figure S1). Moreover, reduction in body weight was greater during the first period and diminished in the second part of the study, regardless of the dietary sequence (Figure S1). Changes in other biometric measures such as BMI, waist to hip ratio and fat to lean mass ratio lipids, blood pressure and in gut microbiome composition (Shannon and Simpson indexes) were also similar after the two diets (Figure S1).

### 3.1. Improvement in Insulin Resistance and HOMA-IR

In order to investigate whether an improvement in insulin resistance could be achieved after the two diets, we compared elicited effects on insulin and HOMA-IR variation using linear mixed effect regression models, with adjustment for treatment period and intervention sequence.

**Figure 2:**
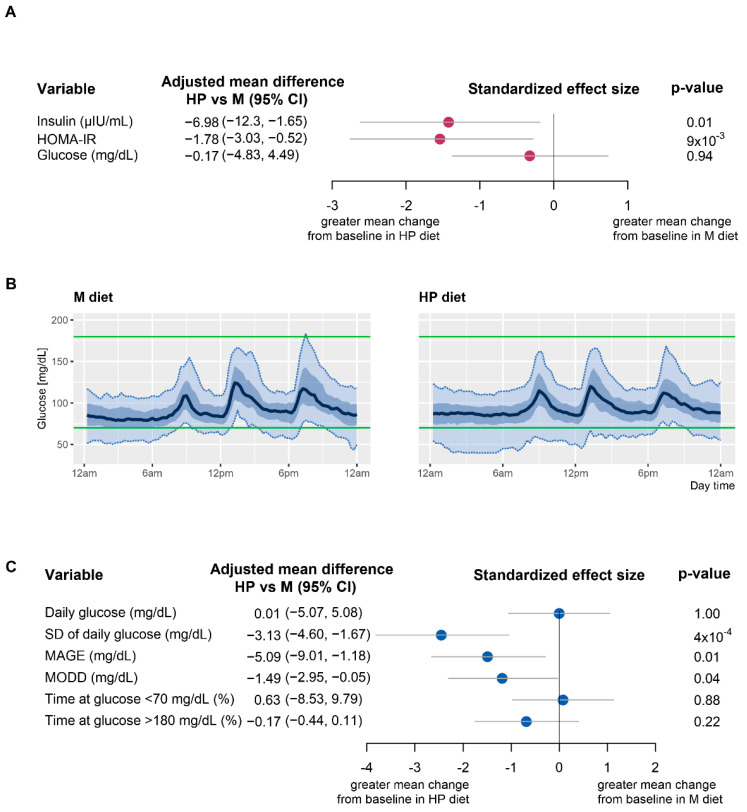
(**A**) Effect of diet on the mean change from baseline of insulin, HOMA-IR and glucose. Mean differences between high protein and Mediterranean diet in change from baseline of considered variables along with related standardized effect size and *p*-values are shown. Estimates were calculated using a mixed effect regression model, after adjusting for dietary sequence and treatment period. (**B**) Summary sensor glucose profiles for 24 h during (i) Mediterranean) and (ii) high protein diet. The solid blue line represents the median, the dark-blue shaded areas the interquartile range, while the light-blue shaded areas the 5th–95th percentile range. Green lines represent the normal range of glucose concentration, 70–180 mg/dL. (**C**) Effect of diet on glucose-related outcomes. Mean difference in glucose-related indices between high protein and Mediterranean diets along with related standardized effect size and *p*-values are shown. Estimates were calculated using a mixed effect regression model, after adjusting for dietary sequence and treatment period. HP: high protein diet, M: Mediterranean diet; HOMA-IR: homeostasis model assessment of insulin resistance CI: confidence interval; SD: standard deviation; MAGE: mean amplitude of glycemic excursions; MODD: mean of daily differences; (*N* = 16).

As shown in Figure 2A, the HP diet was more effective in reducing insulin levels, leading to a mean change from baseline of −3.50 (95% CI: −8.22, 1.21) µIU/mL, while higher levels were registered after the M diet with a value of 1.55 (95% CI: −1.08, 4.18) µIU/mL. Similarly, the HP diet led to a greater reduction in HOMA-IR with respect to the M diet with mean change from baseline of −0.996 (95% CI: −2.11, 0.12) and 0.32 (95% CI: −0.32, 0.96). Differences in the two outcomes between diets were statistically significant (*p* = 0.01, *p* = 9 × 10^−3^). Reduction in glucose concentration was slightly greater in HP diet (−2.44 (95% CI: −6.02, 1.14) mg/dL) with respect to M diet (−1.88b (95% CI: −491, 1.16) mg/dL), however the difference between the two interventions was not statistically significant (*p* = 0.55).

### 3.2. Effect of HP and M Diets on Glycemic Variability

To further investigate the possible differential effect of the two dietary regimens on glucose variability, continuous monitoring data on glucose concentration were analyzed. Figure 2B shows 24 h sensor glucose profiles for each diet (individual patients’ profiles are reported in Figure S2). Mean differences between interventions in SD of glucose concentration and other glucose summary outcomes, after adjustment for dietary sequence and treatment period, are presented in Figure 2C along with related standardized effect size and *p*-values (means of glucose outcomes in the two groups are reported in Table S2).

Patients while on HP diet improved glycemic variability, showing a significant reduction in SD of glucose concentration (Figure 2C), with a mean of 14.79 (95% CI: 12.83, 16.75) mg/dL compared to 17.92 (95% CI: 15.96, 19.89) mg/dL observed during M diet (*p* = 4 × 10^−4^ for the difference) as reported in Table S2. Consistent results were also observed for both mean amplitude of glycemic excursions (MAGE) and the mean of daily differences (MODD) [26], Figure 2C. CGM data supported the previous indication that glucose levels were not affected by the type of diet, as the mean daily concentration of blood glucose was comparable in the two groups (Figure 2C).

Patients spent similar sensor time at glucose levels below 70 mg/dL during HP diet and M diet. Time spent in hyperglycemic conditions at glucose levels above 180 mg/dL was limited and comparable for the two diets. No differences were detected between diets in indices of low and high blood glucose risks (data not shown).

### 3.3. Association of Baseline Gut Microbial Composition at Genus Level with Glucose Variability

**Figure 3:**
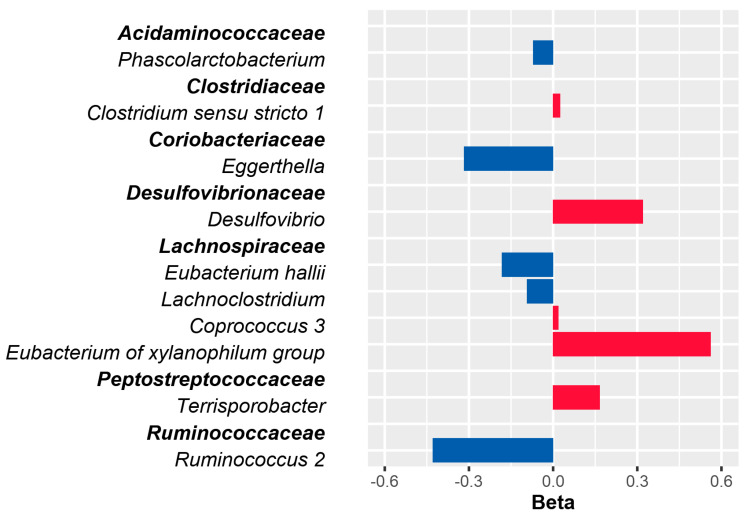
Microbial genera associated with the difference in glucose standard deviation between diets. Association between baseline microbial composition and mean difference of glucose index between HP and M diets was evaluated using zero sum constraint regression model. Results are reported in terms of beta regression coefficients, where the increase in the relative abundance of the selected genus at baseline is associated with an increased difference (red bars) or a decreased difference (blue bars) in SD of glucose concentration between HP and M diets. OTUs at baseline were first agglomerated to genus level. Relative abundances were filtered for sparsity and log transformed before the analysis. SD: standard deviation; (*N* = 16).

We further investigated whether the patients’ gut microbiome composition could be related to the difference in glycemic variability observed in the two diets. After aggregating OTUs into 148 genera, with the use of a zero sum constraint regression model, we identified a panel of 10 microbial genera (Figure 3).

Of the 10 identified microbial taxa, 4 genera were annotated to the family of *Lachnospiraceae* (with opposite directions), one genus to *Ruminococcaceae* (with negative direction), *Peptostreptococcaceae* (with positive direction), *Acidaminococcaceae* (with negative direction), *Clostridiaceae* (with positive direction), *Coriobacteriaceae* (with negative direction) and *Desulfovibrionaceae* (with positive direction) families.

## 4. Discussion

In this 21-day randomized crossover controlled inpatient feeding trial, we found the HP diet to be more effective in reducing insulin resistance and in improving glycemic variability, compared to the M diet in 16 morbidly obese women with pre-diabetes. Moreover, we identified a panel of 10 microbial genera underlying the difference in glycemic variability between the two diets. These include microbes previously associated with the regulation of glucose homeostasis and insulin resistance [25,27].

We have also reported that both diets are equally effective in reducing weight (Figure S1) with participants consistently losing more weight during the first half of the study, compared to the second half. The lack of difference observed in weight, waist to hip ratio and BMI between the M and HP diets suggests that the weight loss observed may be primarily due to the isocaloric nature of the two diets, rather than specific dietary components. Moreover, participants may have benefitted overall from improved nutrition (increased fiber, PUFA, etc.) compared to their previous dietary habits; however, this information was unavailable for study.

Beneficial effects on health outcomes and metabolic functions have been reported in individuals adhering to both HP and M diets [28,29]. A HP diet has been linked with greater improvements in metabolic health and insulin sensitivity in individuals, mainly obese, overweight or insulin-resistant, when compared to alternate diets if weight loss is achieved [30,31]. Indeed, a greater decrease in HOMA-IR/insulin was reported in (i) obese women on an 8-week HP compared to those on a low protein diet [32]; (ii) in obese women on a 9-month isocaloric HP-low carbohydrate diet compared to those adhering to a standard isocaloric diet [33]; (iii) in overweight and obese women with the highest protein uptake in a 6-month calorie reduced diet with increasing protein content (20%, 27% or 35%) [33,34], among others. Our results are consistent and support, in this study group, a greater improvement in insulin resistance and related parameters after following an HP diet.

In our study, HP and M diets did not elicit an effect on the mean glucose level, both during clinical visits and when using 24 h glucose monitoring data. This observation is in line with several studies that reported no effect on the mean glucose levels after high protein intake in T2D patients [35]. However, we found a lower glycemic variability in morbidly obese women following the HP diet. Glucose variability is a risk factor for T2D development and complications [36], and increases in variability may be considered an additional parameter in the assessment of glucose homeostasis at the early stages of glucose dysregulation [37,38]. Reducing glucose variability by diet in non-diabetic patients may be clinically relevant because at early stages of dysglycemia; there is a decline of the cardiac autonomic function that is related to glucose variability and HOMA-IR [39]. In addition, glucose variability is associated with in-hospital complications and longer hospitalization following surgery [40,41,42] and with mortality in critically ill subjects [43].

Several studies have linked glycemic control to gut microbiome composition [36,44,45]. When we investigated the role of gut microbiome composition in our study, we found that *Eubacterium xylanophilum*, *Desulfovibrio*, *Terrisporobacter*, *Clostridium sensu stricto* and *Coprococcus* presented a positive effect on glycemic variability following the HP diet. This suggests that these genera might play an important role in improving host glucose homeostasis. For instance, *Coprococcus* might exert such a positive effect by its production of short-chain fatty acids (SCFA) [46]. Several studies have reported the benefits of SCFA in glucose homeostasis by regulating the blood glucose levels and glucose uptake [46]. Likewise, *Coprococcus* spp. might be able to metabolize the vitamins folate and biotin, which have been associated with lower plasma glucose levels [27]. On the other hand, a negative association was found with *Ruminococcus*, *Eggerthelia*, *Eubacterium hallii*, *Lachnoclostridium* and *Phascolarctobacterium*, suggesting that they might negatively impact glucose metabolism, and thus, type-2 diabetes. We have previously investigated the functional capabilities of *Lachnoclostridium spp*. and reported that *Lachnoclostridium* might metabolize both choline and phosphatidylethanolamine [27], which are precursors of trimethylamine (TMA) and TMA-N-oxide (TMAO), thereby negatively regulating glucose metabolism and insulin sensitivity [37].

Our study benefits from a highly controlled nature; we can be confident in participants’ adherence to the diet and its effect on the observed changes. However, we cannot infer any information about the long-term effects of either diet, as this study was only 21 days long, with participants spending 10 days on each diet. A study over a longer time course may reveal differences between the M and HP diets.

We also note some limitations. Our study has a small sample size with only 20 participants and a drop-out rate of 20%. Second, there was no wash-out period between the two diets. Third, study participants did not do an oral glucose tolerance test. Although the OGTT provides useful information about glucose tolerance, it does not infer on insulin sensitivity/resistance per se [47]. Moreover, under fasting conditions, basal insulin secretion determines a relatively constant level of insulinemia that will be lower or higher in accordance with insulin sensitivity such that hepatic glucose production matches whole body glucose disposal under fasting conditions. Thus, surrogate indexes based on fasting glucose and insulin concentrations, such as HOMA-IR, provide a greater reflection of primarily hepatic insulin sensitivity/resistance. Finally, only 11 participants provided stool samples for the entire duration of the study. Further work to investigate the role of gut microbiome composition and diversity in individual response to dietary intervention would be of considerable interest. Moreover, the study design lacked a wash-out period between dietary crossovers, as dietary effects may be brought about over a longer duration, some crossover effects of the previous diets may have been observed.

## 5. Conclusions

In conclusion, we find that the HP diet is more effective in reducing insulin resistance and in improving glycemic variability in morbidly obese women with pre-diabetes and have identified a panel of 10 microbes underlying the difference in glycemic variability between the two diets. Further investigation is required to elucidate the links between dietary interventions, the microbiome and clinical outcomes, as well as to identify measures that are predictive of individual response to intervention. Continued investigation of these interactions will contribute to the development of stratified intervention and prevention strategies for obesity and its associated health problems.

## Figures and Tables

**Table 1:** Clinical and biochemical characteristics of obese women at baseline.

Phenotype	HP–M	M–HP
*N*	11	5
Sex, *n* (%)	11 (100%)	5 (100%)
Impaired Fasting Glucose, *n* (%)	3 (27.3%)	1 (20%)
Age, years	36.18 (12.55)	42.40 (15.32)
Weight, Kg	118.94 (17.98)	130.86 (38.84)
BMI, Kg/m^2^	44.56 (4.61)	50.40 (10.79)
Waist to hip ratio	0.86 (0.07)	0.91 (0.12)
Fat to lean mass ratio *	1.06 (0.16)	1.28 (0.25)
SBP, mmHg	127.27 (11.04)	129.00 (11.40)
DBP, mmHg	79.09 (8.61)	79.00 (8.94)
Heart Rate, bpm	82.55 (11.71)	78.40 (15.47)
Triglycerides, mmol/L	1.27 (0.34)	1.58 (0.56)
Total cholesterol, mmol/L	4.12 (0.78)	4.65 (0.86)
LDL cholesterol, mmol/L	1.61 (0.43)	1.94 (0.45)
HDL cholesterol *, mmol/L	1.17 (0.11)	1.00 (0.08)
Glucose, mg/dL	93.27 (8.13)	94.80 (6.53)
Insulin, µIU/mL	19.98 (8.28)	24.42 (11.52)
HOMA-IR	4.60 (2.05)	5.61 (2.43)
HbA1c, %	5.55 (0.25)	5.68 (0.44)
Shannon Index	5.76 (0.42)	5.58 (0.37)
Simpson Index	0.96 (0.01)	0.96 (0.01)

Mean (SD). HP: high protein diet; M: Mediterranean diet; BMI: body mass index; SBP: systolic blood pressure, DBP: diastolic blood pressure, LDL: low-density lipoprotein; HDL: high-density lipoprotein; HOMA-IR: homeostasis model assessment of insulin resistance; HbA1c: hemoglobin A1c; * Statistically significant difference between two sequence groups according to *t*-test (fat to lean mass ratio: *p* = 0.05; HDL cholesterol: *p* = 0.01).

## Data Availability

Data for this study is deposited on Mendeley (Mendeley Data, V1, doi:10.17632/nsnm9tjrnt.1).

## Supplementary Materials

The following are available online at https://www.mdpi.com/article/10.3390/nu13124380/s1, Table S1. Details on food provided with the two diets. Table S2. Difference between the two diets in glycemic exposure, control and variability. Figure S1. Effect of diet on the mean change from baseline of anthropometric measures, blood pressure, heart rate and lipids. Figure S2. Individual 24 h median sensor glucose profiles according to the type of diet.
