# Supplementary material for: A High Protein Diet Is More Effective in Improving Insulin Resistance and Glycemic Variability Compared to a Mediterranean Diet—A Cross-Over Controlled Inpatient Dietary Study
Source: Nutrients. 2021 Dec 7;13(12):4380. doi: 10.3390/nu13124380 (PMC8707429; doi:10.3390/nu13124380)
Supplement: Supplementary file 1 [file nutrients-13-04380-s001.zip › nutrients-1487721-supplementary.pdf]

Table S1. Details on food provided with the two diets.

Meal	HP diet	M diet
**Breakfast**	Milk or yogurt with white bread or rusks	Milk or yogurt with wholemeal bread or wholemeal rusks
**Lunch/Dinner**	First course: pasta or white rice with simple seasoning (vegetables or tomato sauce) and Parmesan cheese;<br>Second course*: white meat, fish, eggs, legumes or cheese and seasonal vegetables with olive oil and wholemeal bread;<br>Fruit flavoured low-fat yogurt or fresh fruit	First course: wholemeal pasta with simple seasoning (vegetables or tomato sauce);<br>Second course †: white meat, bluefish, eggs, legumes or goat cheese and seasonal vegetables with extra virgin olive oil and wholemeal bread;<br>Fresh fruit
**Afternoon snack**	-	Walnuts

* Second course protein source: in HP diet was: 4/14 meals with white meat, 4/14 with fish, 2/14 with eggs, 2/14 with legumes and 2/14 with cheese. † Second course protein source in M diet was: 3/13 meals with white meat, 3/14 with bluefish, 4/14 with legumes, and 2/14 with goat cheese and 2/14 with eggs.

Table S2. Difference between the two diets in glycemic exposure, control and variability.

Glucose outcome	N	Diet	Unadjusted mean (95% CI)	Unadjusted difference HP vs M diet (95% CI)	Adjusted difference HP vs M diet (95% CI)	p
Glucose exposure						
**Mean daily glucose concentration (mg/dl)**	16	HP	93.15 (84.01, 102.28)	-0.29 (-4.82, 4.25)	0.006 (-5.07, 5.08)	0.998
		M	93.43 (86.71, 100.15)			
Glucose variability						
**Standard Deviation of daily glucose concentration (mg/dl)**	16	HP	14.80 (13.19, 16.42)	-2.17 (-4.02, -0.33)	-3.13 (-4.60, -1.67)	0.0004
		M	16.97 (14.70, 19.25)			
**Mean Amplitude of Glucose Excursion**	16	HP	29.58 (26.26, 32.90)	-2.87 (-7.49, 1.75)	-5.10 (-9.01, -1.18)	0.014
		M	32.45 (28.20, 36.70)			
**Mean Of Daily Differences**	16	HP	10.67 (9.29, 12.06)	-0.64 (-2.38, 1.11)	-1.50 (-2.95, -0.048)	0.044
		M	11.31 (10.13, 12.49)			
Glucose ranges						

**Proportion of time spent with glucose level in range 70–180 mg/dl (%)**	16	HP<br>M	86.91<br>(75.13, 98.69)<br>88.93<br>(81.23, 96.63)	-2.02<br>(-10.17, 6.13)	-0.77<br>(-9.72, 8.18)	0.8568
**Proportion of time spent with glucose level <70 mg/dl (%)**	16	HP<br>M	13.63<br>(1.46, 25.80)<br>11.91<br>(3.82, 20.01)	1.72<br>(-6.57, 10.01)	0.63<br>(-8.53, 9.79)	0.885
**Proportion of time spent with glucose level >180 mg/dl (%)**	16	HP<br>M	0.23<br>(0.06, 0.40)<br>0.31<br>(0.08, 0.54)	-0.08<br>(-0.35, 0.19)	-0.17<br>(-0.44, 0.11)	0.223

Adjusted estimates with their 95% CI were calculated by a mixed effect regression model. Fixed terms were treatment (HP over M), sequence, period (P1 over P2). HP: high protein diet; M: Mediterranean diet; (N=16).
